# Translational Regulation Promotes Oxidative Stress Resistance in the Human Fungal Pathogen Cryptococcus neoformans

**DOI:** 10.1128/mBio.02143-19

**Published:** 2019-11-12

**Authors:** Jay Leipheimer, Amanda L. M. Bloom, Christopher S. Campomizzi, Yana Salei, John C. Panepinto

**Affiliations:** a Department of Microbiology and Immunology, Witebsky Center for Microbial Pathogenesis and Immunology, University at Buffalo, SUNY, Buffalo, New York, USA; b Department of Biochemistry, University at Buffalo, SUNY, Buffalo, New York, USA; c Charles E. Schmidt College of Medicine, Florida Atlantic University, Boca Raton, Florida, USA; Duke University Medical Center

**Keywords:** *Cryptococcus neoformans*, mRNA degradation, mRNA stability, oxidative stress, stress response, transcription factors, transcriptional regulation, translational control

## Abstract

Cryptococcus neoformans is one of the few environmental fungi that can survive within a mammalian host and cause disease. Although many of the factors responsible for establishing virulence have been recognized, how they are expressed in response to certain host-derived cellular stresses is rarely addressed. Here, we characterize the temporal translational response of C. neoformans to oxidative stress. We find that translation is largely inhibited through the phosphorylation of the critical initiation factor eIF2α (α subunit of eukaryotic initiation factor 2) by a sole kinase. Preventing eIF2α-mediated translational suppression resulted in growth sensitivity to hydrogen peroxide (H_2_O_2_). Our work suggests that translational repression in response to H_2_O_2_ partly facilitates oxidative stress adaptation by accelerating the decay of abundant non-stress-related transcripts while facilitating the proper expression levels of select oxidative stress response factors. Our results illustrate translational suppression as a critical determinant of select mRNA decay, gene expression, and subsequent survival in response to oxidative stress.

## Abstract (precis)

Fungal survival in a mammalian host requires the coordinated expression and downregulation of a large cohort of genes in response to cellular stresses. Initial infection with C. neoformans occurs in the lungs, where it interacts with host macrophages. Surviving macrophage-derived cellular stresses, such as the production of reactive oxygen and nitrogen species, is believed to promote dissemination into the central nervous system. Therefore, investigating how an oxidative stress-resistant phenotype is brought about in C. neoformans not only furthers our understanding of fungal pathogenesis but also unveils mechanisms of stress-induced gene reprogramming. We discovered that H_2_O_2_-derived oxidative stress resulted in severe translational suppression and that this suppression was necessary for the accelerated decay and expression of tested transcripts.

## INTRODUCTION

Cryptococcus neoformans, an encapsulated fungus that causes meningitis and respiratory infection in both immunocompetent and immunocompromised individuals, is estimated to affect 220,000 people annually (1). In the context of a human host, M1 macrophage activation has been found to be essential for fungal killing, which is believed to be mediated through the production of reactive oxygen and nitrogen species (ROS and RNS, respectively) (2–4). High levels of ROS can cause major disruptions in cellular functions through oxidation of proteins, lipids, and nucleic acids (5). Therefore, oxidants must be contended with quickly and the damage caused by them repaired. Subjecting C. neoformans cultures to hydrogen peroxide (H_2_O_2_), which generates ROS, has been found to induce the simultaneous transcriptional expression of stress response factors coupled with the downregulation of homeostatic mRNAs (6). In Saccharomyces cerevisiae, H_2_O_2_ is met with strong translational inhibition (7). This inhibition was found to be achieved partly through the suppression of active ternary complex, which affects the rate of translation initiation (8). In the cytoplasm, the 5′ ends of mRNA possess a methylated guanosine (cap) that protects it from 5′-3′ exonucleases while the 3′ end is protected from 3′-5′ exonuclease activity by the presence of long tracts of adenines [poly(A) tail] bound by poly(A) binding protein (Pab1p) (9–12). The described elements that protect the mRNA from decay also promote the translation of the transcript. Therefore, according to our current understanding, it seems that the mRNA decay and translational machinery are competing for the same elements found on a transcript. Indeed, an inverse correlation has been found between an mRNA’s translation initiation rate and its half-life, suggesting that one predominates over the other under certain conditions (13).

For the first time, in C. neoformans, we have characterized the translational response to oxidative stress. We find that translation is severely inhibited in response to H_2_O_2_ in an eIF2α (α subunit of eukaryotic initiation factor 2)-dependent manner. We used puromycin incorporation and polysome profiling to show that oxidative stress does not result in complete translational inhibition and that many oxidative stress response mRNAs are able to associate with ribosomes. Our work supports translational inhibition as the driving force of initial oxidative stress-induced decay of transcripts abundant under unstressed conditions, with eIF2α phosphorylation triggering the decay. Importantly, translational repression is a requirement for oxidative stress resistance and can be conferred by carbon starvation in an eIF2α-independent manner. Altogether, this work characterizes the interplay between mRNA translation and decay as it pertains to oxidative stress resistance in a human fungal pathogen.

## RESULTS

### Translation is temporally inhibited in response to oxidative stress in a dose-dependent manner.

**FIG 1:**
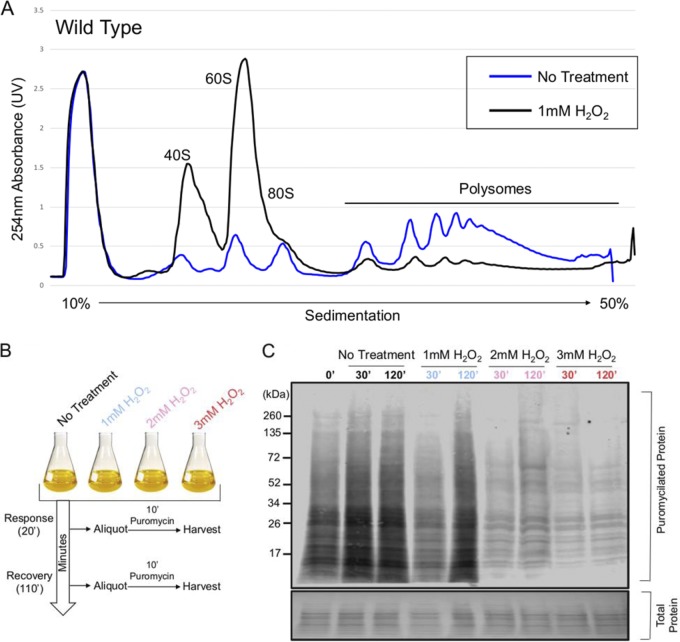
Translation is rapidly repressed in response to hydrogen peroxide-induced oxidative stress. (A) Cultures were grown to exponential phase in YPD at 30°C before incubation in the presence or absence of 1 mM H_2_O_2_ for 30 min. Absorption peaks derived from the lower portion of the gradient represent rRNA making up ribosomes bound to mRNA. Higher peaks in the heavy portion (polysomal) of the gradient compared to the lighter portion (subpolysomal) under the untreated condition indicate that most ribosomes at this point are engaged in translation. This pattern is reversed upon treatment with H_2_O_2_, with many of these ribosomes dissociating from mRNA and instead sedimenting with the subpolysomal fraction. (B) Visual representation of experimental strategy for the assay shown in panel C. Cultures were subjected to various concentrations of H_2_O_2_ with translational output assessed at two time points. (C) Visualization of puromycin incorporation in cultures exposed or not to various concentrations of H_2_O_2_ at 30°C for either 30 or 120 min. Puromycin incorporation was detected by Western blotting with an antipuromycin secondary antibody. Equivalent loading is indicated by the total protein stain.

In the ascomycete S. cerevisiae, translation is inhibited in response to the exogenous addition of H_2_O_2_ to the culture medium, and this inhibition is crucial for oxidative stress-induced damage recovery (8). To determine the global translational state of the basidiomycete C. neoformans in response to oxidative damage, we chose to observe ribosome activity using two approaches. To compare the concentrations of mRNA bound to free ribosomes, polysome profiles were derived from cultures grown to exponential phase at 30°C (unstressed) and after 30 min of exposure to 1 mM H_2_O_2_. Polysome profiling, which examines the extent of ribosome association with mRNA in lysate by separating out large macromolecular complexes based on density in a sucrose gradient subjected to ultracentrifugation, suggests that the majority of the C. neoformans 40S and 60S subunits are engaged with mRNAs at exponential phase (Fig. 1A). However, in response to H_2_O_2_, most of these ribosomes dissociate from mRNA and instead are found in the less dense portion of the gradient. A profile of this nature strongly suggests that translational output is low and that many of the free ribosome subunits are unable to associate with mRNA due to either a lack of active initiation factors or possibly a decrease in the total translatable mRNA.

Despite severe translational suppression in response to H_2_O_2_, a subset of mRNAs remain associated with ribosomes in the heavy polysome fraction. We, however, could not assume that these ribosomes are actively decoding the bound mRNA, as prior studies report that translational elongation may be the target of inhibition in response to oxidative stress (14). Therefore, we probed the translational output of C. neoformans in response to various concentrations of H_2_O_2_, *in vivo*, using puromycin incorporation as a readout. Puromycin, which is an aminonucleoside antibiotic produced by the bacterium Streptomyces alboniger, covalently binds to the growing nascent polypeptide chain during active translation (15). Higher overall rates of translational elongation and numbers of ribosomes engaged in elongation result in higher incorporation of puromycin, which can be detected in immunoblot assays using an antibody to the compound (see Fig. S1 in the supplemental material). Because puromycin can be incorporated at any point along any transcript during elongation, the resulting molecular weights are dispersed throughout the Western blot lane. Puromycin was added to the culture medium 10 min prior to the indicated harvest time to limit any detrimental effects that puromycin may have on growth (Fig. 1B). The extent of translational repression corresponded to the concentration of H_2_O_2_ used in the experiment, with larger amounts causing greater repression (Fig. 1C). Likewise, increasing concentrations of H_2_O_2_ extended the length of time that cultures spent in a translationally repressed state. Therefore, these results indicate that C. neoformans is able to regulate the extent of translational inhibition in response to the severity of the oxidative stress. However, translation is not completely inhibited even in the presence of higher levels of oxidative stress, suggesting that a subset of mRNAs and ribosomes are resistant to translational repression induced by H_2_O_2_.

10.1128/mBio.02143-19.1 FIG S1 Experimental validation of the use of puromycin in C. neoformans to assess translational output. Yeast were grown to mid-log phase in minimal medium supplemented with 2% dextrose. At that time, cultures were harvested in the presence of either puromycin, cycloheximide, or both for the indicated amount of time. The image on the left represents total protein loaded into each lane following membrane transfer. Signal intensity on the right is derived from immunoblotting against nascent proteins covalently bound with puromycin during translation. Detection of nonspecific binding of the antipuromycin antibody is low (lane 1). Cycloheximide was used as a negative control to assess the need for translational elongation for puromycin incorporation. Download FIG S1, TIF file, 1.2 MB. Copyright © 2019 Leipheimer et al. 2019 Leipheimer et al. This content is distributed under the terms of the Creative Commons Attribution 4.0 International license.

### Oxidative stress-induced translational repression is driven by the phosphorylation of eIF2α and is required for oxidative stress resistance.

**FIG 2:**
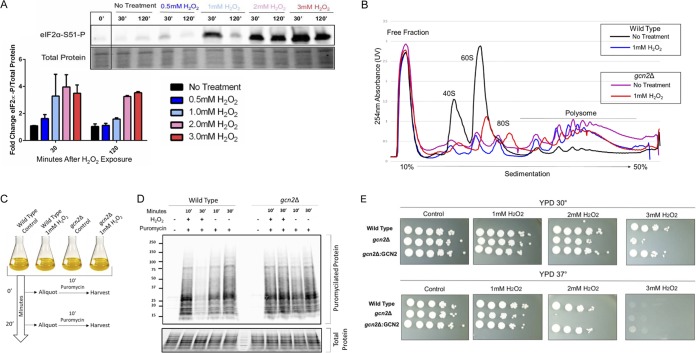
eIF2α is phosphorylated by Gcn2 upon exposure to hydrogen peroxide in a dose-dependent manner. (A) Phosphorylation status of eIF2α was assessed by Western blotting analysis using an antibody recognizing the phosphorylated form of the epitope. Cultures were grown to mid-log phase in minimal medium supplemented with 2% dextrose and were then treated with the indicated concentrations of H_2_O_2_. Cultures were harvested 30 and 120 min after treatment. Time point 0 represents cultures prior to exposure and was used to determine the fold change in phosphorylation. Equivalent loading is indicated by the total protein stain. *n* = 3. (B) Cultures were grown to exponential phase in YPD at 30°C before incubation in the presence or absence of 1 mM H_2_O_2_ for 30 min as done in Fig. 1A. Profiles generated in the *gcn2*Δ strain were overlapped with results from the wild type for comparison. *n* = 4. (C) Schematic highlighting experimental treatment prior to lysis. Cultures were grown to mid-log phase in minimal medium supplemented with 2% dextrose before treatment with or without 1 mM H_2_O_2_. Puromycin was added 10 min prior to harvest at indicated times. (D) Puromycilated proteins are visible by Western blotting analysis using an antibody to the covalently bound antibiotic. No signal was detected in cultures that were not subjected to puromycin. Equivalent loading is indicated by the total protein stain. Numbers at left are molecular masses in kilodaltons. (E) Serial dilution assays were performed by suspending cultures grown for 16 h at 30°C to an OD_600_ of 1.0 and serially diluting them 10-fold in a 96-well plate. Five microliters of the diluted yeast suspension was then placed on YPD imbued with various concentrations of H_2_O_2_. Plates were then incubated at either 30°C or 37° for 2 to 3 days.

We next aimed to discover the underlying mechanism, in C. neoformans, that facilitates such rapid and severe translational suppression in response to oxidative stress. Translation is partly regulated at the level of initiation, which is considered the rate-limiting step of protein synthesis. A major molecular strategy used by eukaryotes in response to a variety of stresses is to limit the availability of functional initiator tRNA ternary complex, which is required for subunit joining at the canonical start codon (16). Phosphorylation of the α subunit within the eIF2 complex at a conserved serine position prevents the recycling of new initiator tRNA following prior delivery to the start codon, thereby, preventing translation initiation through canonical means (17). To assess the status of eIF2α in response to oxidative stress in C. neoformans, we performed immunoblotting using an antibody that recognizes the phosphorylated form of eIF2α. Increasing concentrations of hydrogen peroxide resulted in increased levels of overall phosphorylation of eIF2α following a 30-min exposure (Fig. 2A). Whereas levels of eIF2α phosphorylation return to basal levels in cultures exposed to lower concentration of H_2_O_2_ by 2 h postexposure, subjecting cultures to 2 and 3 mM concentrations resulted in sustained levels of eIF2α phosphorylation. The extent of eIF2α phosphorylation correlated well with the degree of translational repression seen in the above-described puromycin incorporation assays (Fig. 1C).

To assess the importance of eIF2α phosphorylation in promoting translational repression in response to oxidative stress, we sought to eliminate eIF2α kinase activity in C. neoformans. Sequence comparisons of known eIF2α kinases suggested that C. neoformans may possess only one candidate eIF2α kinase with homology to Gcn2, which, of the eIF2α kinase family members, has the widest distribution among eukaryotes (18). Ablating the gene encoding Gcn2 in C. neoformans (CNAG_06174) resulted in the absence of any observable eIF2α phosphorylation signal after exposure to H_2_O_2_ (Fig. S2). Having observed that levels of eIF2α phosphorylation tightly correlate with levels of translational repression and that Gcn2 is required for this phosphorylation, we next performed polysome profiling in the *gcn2*Δ strain (Fig. 2B). Compared to profiles derived from wild-type lysate, where the higher peaks corresponding to the polysome fraction (mRNAs bound to two or more ribosomes) were reduced in response to H_2_O_2_, there was little ribosome dissociation in the absence of Gcn2. These results strongly suggest that ROS-induced translational inhibition in C. neoformans is largely triggered by eIF2α phosphorylation (Fig. S3). It should be noted, however, that a minor decrease in the polysome peaks suggests that eIF2α-independent means of translational suppression are still active. This repression could possibly be brought about by other kinases that affect translation initiation, such as Tor1, or regulation at the level of translation elongation (19–21). Puromycin incorporation assays also showcase the absence of severe translational repression in response to H_2_O_2_ in the *gcn2*Δ strain (Fig. 2C and D). Overall puromycilation was drastically reduced in wild-type yeast 30 min following peroxide treatment compared to untreated conditions, whereas overall puromycilation was not decreased in the *gcn2*Δ strain exposed to the same stress. This further suggests that peroxide-mediated translational inhibition occurs through Gcn2. To address the phenotypic consequence of preventing eIF2α phosphorylation in response to oxidative stress, we performed serial dilution assays using the wild-type strain (H99), the *gcn2*Δ strain, and the complemented strain (Fig. 2E). There were no observable growth sensitivities in the absence of Gcn2 when strains were grown on culture medium alone. However, the presence of the oxidative stressors H_2_O_2_ (Fig. 2E), *tert*-butylhydroperoxide (*t*-BOOH), and nitric oxide (NO^−^) (Fig. S4) resulted in a severe growth sensitivity in the *gcn2*Δ strain that was exacerbated by increased concentrations and incubation temperature. Together, these results indicate that Gcn2 is required for the phosphorylation of eIF2α and that the resulting translational repression in response to H_2_O_2_ promotes oxidative stress adaptation.

10.1128/mBio.02143-19.2 FIG S2 Gcn2 is the sole kinase of eIF2α in C. neoformans. Yeast were grown to mid-log phase in YPD. Cultures were then exposed to 1 mM H_2_O_2_ for 30 min to induce eIF2α phosphorylation. Western blot analysis was performed using an antibody that recognizes the phosphorylated form of eIF2α. The bottom panel represents the homology between the N terminus of eIF2α in C. neoformans and that in S. cerevisiae, with the targeted serine outlined in red. An identity of 92% exists between the first 100 amino acids. Download FIG S2, TIF file, 1.2 MB. Copyright © 2019 Leipheimer et al. 2019 Leipheimer et al. This content is distributed under the terms of the Creative Commons Attribution 4.0 International license.

10.1128/mBio.02143-19.3 FIG S3 Hydrogen peroxide and carbon starvation result in translational suppression. Yeast were grown to mid-log phase in minimal medium supplemented with 2% dextrose. Cultures were then pelleted by centrifugation and were resuspended in minimal medium either with or without 2% dextrose in the presence of 1mM H_2_O_2_. Cultures were harvested after 30 min, and polysome profiles were obtained. Following trace acquisition, the areas under the curves corresponding to the polysome and monosome peaks were quantified. The extent of translational suppression was determined by comparing the total polysome peak area to that of the monosome peak area. Error bars represent SDs from three biological replicates, with exact numerical values depicted on the right. One-way nonparametric analysis (Kruskal-Wallis test) determined the results significant with a wild-type strain *P* value of 0.0036 and a *gcn2*Δ strain *P* value of 0.05. Download FIG S3, TIF file, 1.2 MB. Copyright © 2019 Leipheimer et al. 2019 Leipheimer et al. This content is distributed under the terms of the Creative Commons Attribution 4.0 International license.

10.1128/mBio.02143-19.4 FIG S4 Gcn2 is required for growth in the presence of both RNS and ROS. Strains were grown in YPD for 16 h prior to serial dilution onto agar plates containing minimal medium and 2% dextrose imbued with the indicated stressor. To investigate the scope of oxidative stress sensitivity in the absence of Gcn2, *tert*-butylhydroperoxide (*t*-BOOH) and sodium nitrite (NaNO_2_) were used to subject cultures to ROS and RNS, respectively. The pH of the agar containing NaNO_2_ was titrated to 4 to allow the dissociation of NO^−^ into the medium. Plates were incubated at either 30°C or 37°C for 3 days. Download FIG S4, TIF file, 1.2 MB. Copyright © 2019 Leipheimer et al. 2019 Leipheimer et al. This content is distributed under the terms of the Creative Commons Attribution 4.0 International license.

### Preventing eIF2α phosphorylation following oxidative stress results in dysregulation of oxidative stress response transcript levels.

**FIG 3:**
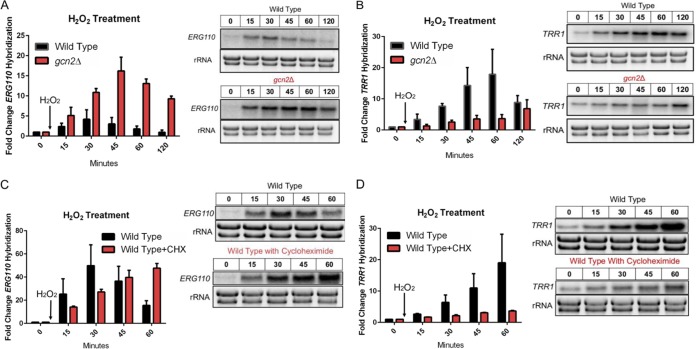
Gcn2-mediated translational inhibition facilitates the proper expression of oxidative stress response transcripts *TRR1* and *ERG110.* Cultures were grown to exponential phase in YPD and were treated with 1 mM H_2_O_2_. Aliquots were harvested at indicated time points, and whole RNA was extracted. Northern blot analysis was performed probing for either *ERG110* (A and C) or *TRR1* (B and D). The rRNA bands were imaged using SYBR Safe stain prior to membrane transfer and were used both as a loading control and to assess RNA sample integrity. (A and B) Steady-state levels of *ERG110* (*P* = 0.0185) and *TRR1* (*P* = 0.0278) were assessed over a 2-h time point following exposure to H_2_O_2_. A representative Northern blot image is shown to the right of the corresponding bar graph. *n* = 3. (C and D) Steady-state levels of *ERG110* (*P* = 0.0363) and *TRR1* (*P* = 0.0199) were assessed over a 1-h time point following exposure to H_2_O_2_ alone or with the addition of translation elongation inhibitor cycloheximide (CHX). *n* = 2. An unpaired *t* test was used to determine if the mean differences between transcript levels of wild-type and *gcn2*Δ strains were statistically significant. Error bars indicate SD between replicates.

Experiments performed in model yeast and other eukaryotes suggest that limiting ternary complexes through eIF2α phosphorylation can favor noncanonical translation of certain transcripts in response to stress, such as those possessing upstream open reading frames (uORF) (17, 22–24). In C. neoformans, many of the ROS response transcripts are predicted to possess extensively structured 5′ untranslated regions (UTR) with uORF, such as the oxidative stress response transcript *ERG110*, which would prevent the recognition of the annotated ORF (6, 25). We hypothesized that preventing eIF2α from being phosphorylated in response to oxidative stress would translationally disfavor *ERG110*. While testing this hypothesis, we were surprised to find that the radioactive signal from the Northern blots probed for the *ERG110* transcript was much higher in the *gcn2*Δ strain than in the wild type (Fig. 3A). It was immediately evident that, in the absence of Gcn2, there is an overabundance of *ERG110* mRNA present compared to the wild-type strain. Furthermore, in comparison to the wild type, where *ERG110* transcript levels return to prestress exposure levels following the initial response to H_2_O_2_, *ERG110* remains abundant in the *gcn2*Δ strain.

The response to severe levels of hydrogen peroxide has previously been described as biphasic, with rapid induction of factors that reduce the cellular environment followed by the expression of factors that repair the damage caused by ROS (26). Thioredoxin reductase (*TRR1*) is an essential gene in C. neoformans responsible for reducing thioredoxin peroxidase (*TSA1*) as well as reducing enzymes responsible for synthesizing basic cellular components required for DNA damage repair, such as ribonucleotide reductase (27, 28). *TRR1* is induced in response to H_2_O_2_, and levels remain high throughout the experimental time points (Fig. 3B). However, in the absence of Gcn2, levels of *TRR1* are well below what is observed in the wild type. The defect in *TRR1* levels does not seem to be due to a defect in the expression of the transcript’s respective transcription factor, as *ATF1* levels are higher than expected in the *gcn2*Δ strain compared to the wild type (29) (Fig. S5A). It is important to note that not all transcripts are dysregulated in the absence of Gcn2, as levels of *TSA1*, which acts to reduce H_2_O_2_ and indirectly promote the expression of *TRR1*, were found to be equivalent under time points tested (30, 31) (Fig. S5B).

10.1128/mBio.02143-19.5 FIG S5 Expression of transcripts associated with *TRR1* transcript induction is still intact in the absence of Gcn2. Cultures were grown to exponential phase in YPD and were subjected to 1 mM H_2_O_2_. Aliquots were harvested at indicated time points during incubation, whole RNA was extracted, and Northern blotting assays were performed probing for *ATF1* (A) and *TSA1* (B). Download FIG S5, TIF file, 0.9 MB. Copyright © 2019 Leipheimer et al. 2019 Leipheimer et al. This content is distributed under the terms of the Creative Commons Attribution 4.0 International license.

Together, these results suggest that the absence of Gcn2 results in the dysregulation of certain stress response genes. To see if this dysregulation is related to the *gcn2*Δ strain’s inability to effectively clear mRNAs of ribosomes immediately following ROS-derived stress, we repeated the prior experimental procedure but with the addition of the translation elongation inhibitor cycloheximide (Fig. 3C and D). Preventing ribosome transcript runoff in response to hydrogen peroxide using cycloheximide in the wild-type strain recapitulated the observed dysfunctional transcript levels observed in the *gcn2*Δ strain for both *ERG110* (Fig. 3C) and *TRR1* (Fig. 3D). These results suggest that polysomal collapse in response to oxidative stress seems to have an effect on the expression of oxidative stress response transcripts, which may stem from the availability of free ribosome subunits.

### Gcn2 is required for the accelerated decay of the “growth-related” transcript *RPL2*.

**FIG 4:**
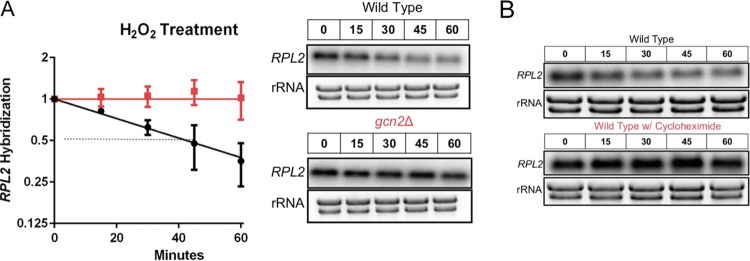
eIF2α phosphorylation is required for the accelerated decay of *RPL2* in response to H_2_O_2_. Cultures were grown to exponential phase in YPD and were subjected to 1 mM H_2_O_2_ under all experimental conditions. (A) A 250-μg/ml concentration of 1,10-phenanthroline was used at the start of the time course to inhibit transcription, allowing for the independent assessment of stability. Whole RNA was extracted, and Northern blot analysis was performed probing for *RPL2*. A representative Northern blot image is shown to the right of the corresponding decay curve. *n* = 3. (B) Representative Northern blot image steady-state levels of *RPL2* (*P* < 0.0001) following exposure to 1 mM H_2_O_2_ with or without the addition of the elongation inhibitor cycloheximide. *n* = 3. Statistical analyses for stability data were obtained by determining the least-squares fit of one-phase exponential decay nonlinear regression. Numbers above blots are time in minutes.

Previous results in our lab have shown that many ribosomal protein (RP) transcripts undergo rapid decay in response to a variety of stresses (32–34). Having observed a defect in the expression of certain stress response transcripts, we asked if the accelerated decay of factors related to ribosome biogenesis in response to stress was also disrupted in the *gcn2*Δ strain. Northern blot analysis was performed probing for the large ribosome protein subunit 2 (*RPL2*) transcript following 1,10-phenanthroline-mediated transcriptional shutoff and H_2_O_2_ exposure (Fig. 4A). Where the half-life of *RPL2* is found to be around 40 min in the wild-type strain, the absence of Gcn2 resulted in a dramatic increase in stability with an unobtainable half-life under the observed time points. To see if the observed defect in *RPL2* transcript reduction was due to ribosomes remaining associated with mRNA in the *gcn2Δ* strain, we again isolated total RNA from the wild-type strain following exposure to H_2_O_2_ and cycloheximide (Fig. 4B). The drastic reduction in the levels of *RPL2* in response to H_2_O_2_ is completely mitigated by the simultaneous addition of the translation elongation inhibitor. These results suggest that, at least for our represented endogenous RP transcript, ribosome dissociation in response to peroxide stress may trigger the rapid decay of certain transcripts and that peroxide stress leads to clearance of these mRNAs from the translational machinery.

**FIG 5:**
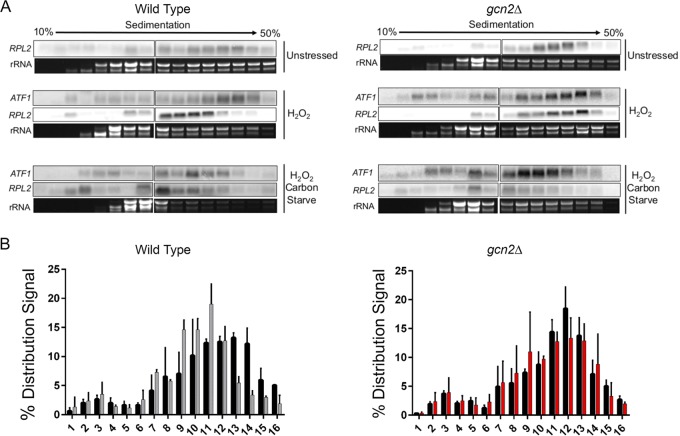
Gcn2 is required for the translational suppression of *RPL2* in response to hydrogen peroxide but not carbon starvation. (A) RNA was isolated and precipitated from fractions acquired following polysome profiling as described in the legend to Fig. S3. All fractions were dissolved in the same volume of water, and a third of that volume was used for Northern blot analysis probing for either *RPL2* or *ATF1.* Prior to membrane transfer, rRNA was visualized and imaged to assess RNA stability and overall distribution through the differential sucrose gradient. Images represent results from three biological replicates. (B) The hybridized signal intensity of *RPL2* in the wild-type and *gcn2Δ* strains under unstressed and stressed conditions was determined for each fraction and summed. The intensity of each respective fraction compared to the total was used to quantify overall distribution of the signal throughout the gradient. Three biological replicates were performed. Error bars indicate SDs.

To determine if the increase in *RPL2* stability in the absence of Gcn2 was directly related to the translational state of that transcript, RNA was isolated from sucrose gradients following ultracentrifugation (Fig. 5A). A transcript is considered highly translated if it is found more so in the high-density portion of the gradient; in contrast, a transcript is considered poorly translated if found distributed in the lower-density portion. Under unstressed conditions, the distribution of *RPL2* in both the wild-type and *gcn2*Δ strains is found in the higher-density portion of the gradient (Fig. 5A, top panel). Whereas exposure to H_2_O_2_ in the wild-type strain resulted in the translational suppression of *RPL2* as observed by a shift in the distribution of the transcript to the lower-density portion of the gradient, the translational state of *RPL2* remained unchanged in the absence of Gcn2 (Fig. 5A, middle panel, and Fig. 5B). This further supports translational suppression as a method for accelerated decay in response to oxidative stress in C. neoformans.

### Glucose starvation results in eIF2α-independent translational suppression and rescues ROS sensitivity in the *gcn2*Δ strain.

**FIG 6:**
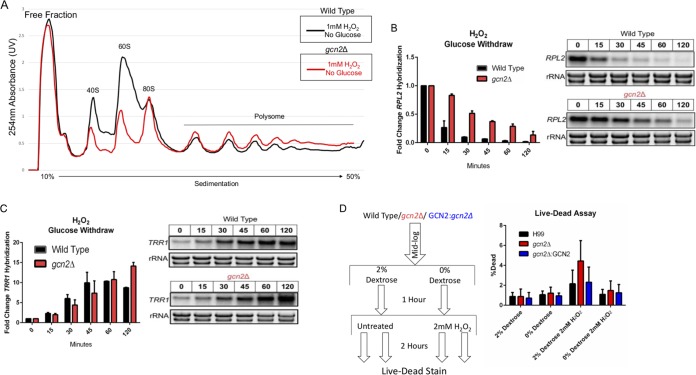
Glucose-mediated translational repression is able to restore oxidative stress resistance in the absence of eIF2α phosphorylation. Cultures were grown to exponential phase in minimal medium supplemented with 2% dextrose (carbon fed), at which point they were pelleted, washed with water, and suspended in minimal medium alone supplemented with 1 mM H_2_O_2_. (A) Polysome profiles were performed using yeast that were harvested and lysed 30 min after resuspension in carbonless minimal medium lacking dextrose with 1 mM H_2_O_2_. (B and C) At indicated time points following resuspension in carbonless minimal medium containing 1 mM H_2_O_2_, whole RNA was extracted and Northern blot analysis probing for steady-state levels of *RPL2* (*P* = 0.0209) or *TRR1* (*P* = 0.8497) was performed. A representative Northern blot image is shown to the right of the corresponding bar graph (*n* = 3). A paired *t* test was used to determine if the mean differences between transcript levels of wild-type and *gcn2*Δ strains were statistically significant. Error bars indicate SDs between replicates. (D) Yeast were grown to mid-log phase in minimal medium supplemented with 2% dextrose. Cultures were then pelleted, washed in water, and resuspended in minimal medium with or without dextrose (carbon starved). After 1 h, cultures were either subjected to 2 mM H_2_O_2_ or left untreated for 2 h prior to Live-Dead staining. The percentage of dead yeast was calculated from the total population determined by thiazole orange staining, which stains all yeast live or dead. *n* = 3.

Preventing ribosome dissociation in response to H_2_O_2_, either through treatment with cycloheximide or by deleting the gene *GCN2*, inhibited both the accelerated decay of *RPL2* and the transcriptional induction of *TRR1*, suggesting that translational repression initiates these events. To challenge this observation, we subjected cultures to carbon starvation, which induced translational suppression through a mechanism independent of eIF2α phosphorylation (Fig. S6A). The translational state of *RPL2* was suppressed to the same extent in the *gcn2*Δ strain as it was in the wild type in response to carbon starvation. To determine if carbon starvation-induced translational suppression could rescue transcript expression in response to oxidative stress in the *gcn2*Δ strain, we subjected cultures to the two conditions simultaneously. Although the addition of H_2_O_2_ did not prevent carbon starvation-mediated suppression in the *gcn2*Δ strain, it was not able to result in the same level of suppression seen in the wild type (Fig. 6A and Fig. S3). This suggests that the two stresses may act on separate translational suppression pathways, with the *gcn2*Δ strain unable to respond to H_2_O_2_ signals for translational suppression.

10.1128/mBio.02143-19.6 FIG S6 Carbon starvation-mediated translational suppression is still intact in the absence of Gcn2. Cultures were grown to exponential phase in minimal medium supplemented with 2% dextrose (carbon fed). Cultures were then pelleted and resuspended in either the same medium or medium lacking dextrose (carbon starved). (A) Cultures were harvested after 30 min, and polysome profiles were obtained. (B) Fractions were then collected sequentially following polysome trace acquisition. RNA was extracted from these fractions, and Northern blot analysis was performed probing for *RPL2* to determine the translational status of the transcript (*n* = 2). Download FIG S6, TIF file, 1.5 MB. Copyright © 2019 Leipheimer et al. 2019 Leipheimer et al. This content is distributed under the terms of the Creative Commons Attribution 4.0 International license.

To further test our earlier hypothesis, that preventing ribosome association with certain mRNAs in response to ROS is needed for both the proper removal and expression of transcripts, we performed a time course analysis of total RNA following the simultaneous removal of glucose and addition of H_2_O_2_. Carbon starvation partially restored the accelerated decline of *RPL2* in the *gcn2*Δ strain (Fig. 6B). Carbon starvation also restored translational suppression of *RPL2*, as observed in the disruption of the transcripts to the lower-density portion of the gradient (Fig. 5A, bottom panel). Although carbon starvation was unable to rescue *ERG110* expression levels in the absence of eIF2α (Fig. S7), it completely restored the expression of *TRR1* (Fig. 6C). These, along with the complementary experiments shown in Fig. 4 and 5, strongly suggest that rapid but transcript-specific translational inhibition in response to H_2_O_2_ is necessary for the expression of critical oxidative stress response transcripts. To determine if the restored transcript kinetics translated to increased oxidative stress resistance in the *gcn2*Δ strain, cultures were treated with a Live-Dead stain following glucose starvation and exposure to H_2_O_2_ (Fig. 6D, schematic). The percentage of dead yeast was determined by flow cytometry and quantified as a percentage of the total. Removing glucose just 1 h prior to the addition of 2 mM H_2_O_2_ returned the oxidative stress resistance of the *gcn2*Δ strain to wild-type levels (Fig. 6D).

10.1128/mBio.02143-19.7 FIG S7 Glucose starvation fails to suppress H_2_O_2_-induced *ERG110* levels in the absence of Gcn2. Cultures were grown to mid-log phase in minimal medium supplemented with 2% dextrose. Cultures were then pelleted, washed with water, and resuspended in carbonless minimal medium with 1 mM H_2_O_2_. Aliquots were harvested, and total RNA was isolated at indicated time points. *ERG110* levels were probed for by Northern blotting analysis (*n* = 2). Download FIG S7, TIF file, 0.8 MB. Copyright © 2019 Leipheimer et al. 2019 Leipheimer et al. This content is distributed under the terms of the Creative Commons Attribution 4.0 International license.

To see if the failure of *TRR1* transcript expression in the *gcn2*Δ strain was due to a defect in the translational expression of the respective transcription factor, *ATF1*, we performed Northern blot analysis against sucrose gradient-fractionated RNA. There were no observed differences in *ATF1* distribution in the polysomes between the two strains (Fig. 5A, middle panel). Furthermore, the distributions of *ATF1* upon carbon starvation and hydrogen peroxide exposure were not different between the strains (Fig. 5A, bottom panel).

## DISCUSSION

The extent and severity of translational repression in response to H_2_O_2_-derived oxidative stress in C. neoformans are driven largely by the phosphorylation of eIF2α. This repression is not absolute, however, as puromycin incorporation is still detected even after being exposed to high levels of H_2_O_2_ (Fig. 1C). Therefore, it seems that a subset of transcripts may possess elements that allow them to be translated under conditions that limit the active ternary complex. Previous examples of mRNAs possessing uORF being translationally favored under conditions of eIF2α phosphorylation are known in other systems (17, 22). One hundred twenty-two predicted uORFs are found to be conserved across four sequenced *Cryptococcus* strains and may represent a major posttranscriptional regulatory strategy for the expression of these transcripts (24). Furthermore, recent (preprint) ribosome profiling results indicate that over a third of C. neoformans transcripts possess uORF that affect translation (35). Our results suggest that certain stressors that activate Gcn2 may translationally favor the expression of the annotated ORF of these transcripts in C. neoformans. Interestingly, the oxidative stress response transcript *TRR1*, which is not expressed in the absence of Gcn2, contains multiple predicted uORF whereas the 5′ UTR of *RPL2*, which is stable in the absence of Gcn2, does not.

Failure to phosphorylate eIF2α resulted in the absence of translational inhibition and the increased stability of *RPL2*. This stability is recapitulated in the wild-type strain upon treatment with cycloheximide, suggesting that ribosome association protects the mRNA from decay factors (Fig. 4B). Promoting translational suppression through glucose starvation in the *gcn2*Δ strain was able to rescue *TRR1* expression and oxidative stress resistance (Fig. 6C and D). How translational suppression restored expression of *TRR1* still remains to be determined. Based on the abundance of the transcription factor *ATF1* and its high polysome association, one would assume that the expression of *TRR1* would be higher than expected, yet the opposite exists. Interestingly, hydrogen peroxide stress has been found to cause a buildup in protein aggregates believed to be caused by protein misfolding (36). Loss of mRNA surveillance pathways that accelerate the decay of certain transcripts further exacerbates protein aggregate formation (37). Could translational suppression favor the proper folding of the transcription factor, and is protein misfolding the true cause of death in C. neoformans exposed to H_2_O_2_? It is an interesting hypothesis given the recent appreciation of cotranslational protein folding (38).

**FIG 7:**
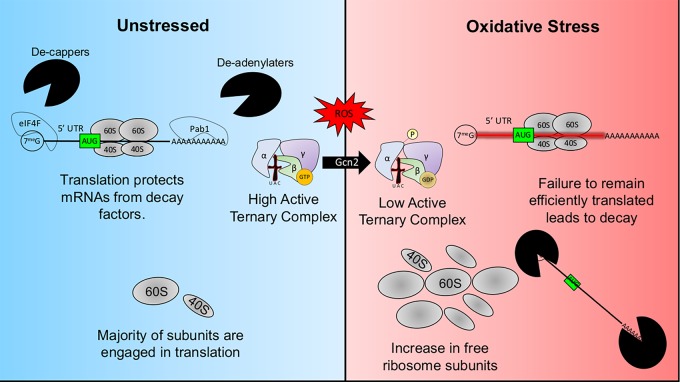
Predicted model of eIF2α phosphorylation-facilitated translational adaptation to ROS. The majority of ribosomes are engaged with translation in yeast grown to exponential phase. The bound ribosome and associated initiation factors compete with the decapping and deadenylating enzymes. ROS-generating factors, however, activate the eIF2α kinase Gcn2 in a manner that has yet to be determined. The extent of Gcn2 activity is proportional to the level of stress and results in the reduction of initiator tRNA ternary complex. This prevents the association of ribosomes with mRNAs that lack the ability to recruit translation factors in an eIF2α manner, and their decay is accelerated. Newly transcribed oxidative stress transcripts now enter a cellular context composed of many free ribosomes, which could enhance the ability to be translated through means that are usually not favored under homeostatic conditions.

Altogether, our results suggest a tight interconnectedness between translation and mRNA decay that drastically affects the ability of the fungal pathogen to adapt to oxidative stress (Fig. 7). Disrupting the translational response to H_2_O_2_ resulted in changes in the stress response at the transcript level indicating the yet-unappreciated role that ribosome availability plays in regulating mRNA levels and ultimately their expression during stress. The scientific community’s understanding of eukaryotic gene regulation has largely been in the context of steady-state exponential-phase growth conditions. Our results suggest that when this context changes, so do the dynamics of gene regulation.

## MATERIALS AND METHODS

### Strains and media.

The strain of Cryptococcus neoformans used in these studies is a derivative of H99O that retains full virulence and melanization. C. neoformans was cultivated on YPD (1% yeast extract, 2% peptone, 2% dextrose) agar unless otherwise indicated. Cultures were grown and seeded at 30°C as previously described (32).

The *gcn2*Δ mutant strain was constructed as described previously (39). Gene deletion and complementation were confirmed by PCR and Northern blot analysis. GCN2 complementation was also confirmed by Western blotting analysis against the phosphorylated form of eIF2α. 5′ UTR reporter constructs were assembled using the NEBuilder HiFi DNA Assembly Cloning kit (catalog no. E5520S; New England Biolabs, Ipswich, MA). All amplifications were carried out according to the manufacturer’s guidelines. pBluescript containing the G418 resistance cassette was used as a vector in the assembly. Full and in-line construct incorporation into the vector was confirmed by sequencing with primers reading into the desired adjacent amplified region. G418-resistant colonies were selected following biolistic transformation, and galactose induction of the mCherry fused reporter was confirmed by Northern blot analysis probing for the mCherry ORF. All oligonucleotide sequences are listed in Table S1 in the supplemental material.

10.1128/mBio.02143-19.8 TABLE S1 Oligonucleotide sequences. Capital letters indicate portion of the primer that hybridizes to the genome. Restriction sites are underlined. Download Table S1, PDF file, 0.3 MB. Copyright © 2019 Leipheimer et al. 2019 Leipheimer et al. This content is distributed under the terms of the Creative Commons Attribution 4.0 International license.

### Isolation of whole-cell lysate.

Whole-cell lysate from yeast cultures was obtained by glass bead (catalog no. 9831; RPI)-mediated mechanical disruption using Bullet Blender Gold (Next Advance model BB2U-AU) set to power level 12 for 5 min. Lysate for the purpose of Western blot analysis was suspended in buffer containing15 mM HEPES (pH 7.4), 10 mM KCl, 5 mM MgCl_2_, 10 μl/ml HALT protease inhibitor [Thermo Scientific, Mount Prospect, IL]). Crude lysate was then centrifuged at 22,000 relative centrifugal force (rcf) at 4°C for 10 min. The cleared lysate was aliquoted from cellular debris into a new tube. Lysate for the purpose of Northern blot analysis was obtained similarly, with the exception of the buffer used. RLT (catalog no. 79216; Qiagen) was used to inhibit RNA decay during lysis. RNA was then isolated from cleared lysate using the manufacturer’s protocol (catalog no. 74106, RNeasy Mini kit; Qiagen).

### Western blot analysis.

Western blotting assays were performed using a total of 25 μg of total protein derived from lysate and suspended in Laemmli sample buffer (catalog no. 1610737; Bio-Rad). Proteins were separated by gel electrophoresis using Bio-Rad Mini-Protean TGX stain-free 4 to 15% Tris-glycine gels (catalog no. 4568085; Bio-Rad). These gels are embedded with a reagent that fluoresces when bound to a protein. Following gel separation, the fluorescence was analyzed and quantified using the Bio-Rad Gel Doc XR+ imager default settings to verify the equal loading of protein across samples. Nitrocellulose transfer was performed using the Bio-Rad Trans-Blot Turbo and corresponding transfer stacks at the instrument’s default TGX setting (catalog no. 170-4270RTA transfer kit; Bio-Rad). Immunoblotting was performed as previously described (40). Primary antibodies anti-EIF2S1 (ab3215; Abcam), anti-mCherry (rabbit) (catalog no. 600-401-P16), and antipuromycin 12D10 (catalog no. MABE343; Millipore) were applied at 1:1,000 for 12 to 18 h at 4°C.

### Stability assays and Northern blot analysis.

Mid-log-phase cells were grown at 30°C in YPD or minimal medium (yeast nitrogen base [YNB]) supplemented with 2% dextrose until exponential phase for all experimental conditions involving lysate-derived experimental approaches. Yeast cultures were subjected to 1 mM H_2_O_2_. At the same time, transcriptional inhibition was achieved by the addition of 1,10-phenanthroline (catalog no. 131377; Aldrich) (250 μg/ml), and cultures were returned to the incubator at 30°C. Experiments accessing steady-state levels of mRNA were performed similarly, except that the transcriptional inhibitor was not included. Northern blot analysis was performed, and blots were imaged using 5 μg of RNA as previously described (41). The hybridized transcript signal was normalized to rRNA gel bands. The half-life of *RPL2* was determined by nonlinear regression of normalized *RPL2* over time (GraphPad).

### Polysome profiling.

Yeast was grown in a 2-liter baffled flask in YPD with shaking at 250 rpm at 30°C for 5 to 6 h, reaching an optical density at 600 nm (OD_600_) of ∼0.55 to 0.65. Polysome profiles were obtained as described previously (32). Yeast cells were then harvested in the presence of 0.1 mg/ml cycloheximide (catalog no. 66-81-9; Acros Organic) and pelleted immediately at 3,000 rcf for 2 min at 4°C. The yeast pellet was then flash-frozen in liquid nitrogen, resuspended, and washed in polysome lysis buffer (20 mM Tris-HCl [pH 8], 2.5 mM MgCl, 200 mM KCl, 1 mg/ml heparin [catalog no. SRE0027-500KU], 1% Triton X-100, 0.1 mg/ml cycloheximide). Yeast cells were then lysed mechanically by glass bead disruption, resuspended in 500 μl of polysomal lysis buffer, and centrifuged for 10 min at 16,000 × *g* and 4°C to obtain the cytosolic portion of the lysate. Total RNA (250 μg) in a 250-μl total volume was layered on top of the polysome sucrose gradient (10% to 50% linear sucrose gradient, 20 mM Tris-HCl [pH 8], 2.5 mM MgCl, 200 mM KCl, 1 mg/ml heparin, 0.1 mg/ml cycloheximide). Gradients were subjected to ultracentrifugation at 39,000 rpm in an SW-41 rotor at 4°C for 2 h. Following centrifugation, sucrose gradients were pushed through a flow cell using a peristaltic pump, and RNA absorbance was recorded using Teledyne’s UA-6 UV-visible (UV-Vis) detector set at 254 nm. Absorption output was recorded using an external data acquisition device (DataQ). Fractions were then collected following absorption using a Teledyne retriever 500 set to collect 16-drop fractions.

To extract RNA, fractions were suspended in 3 volumes of 100% ethanol and incubated at −80°C for 12 to 16 h. The precipitate was collected via centrifugation at 16,000 × *g* at 4°C for 20 min and resuspended in 250 μl warm RNase-free water followed quickly with the addition of 750 μl TRIzol LS (catalog no. 10296010; Invitrogen). RNA was extracted per the manufacturer’s instructions. Purified RNA was resuspended in 30 μl RNase-free water. A third of this volume of each sample was used in subsequent Northern blot analyses.

### Puromycin incorporation assay.

Yeast cultures were grown to mid-log phase for 5 to 6 h in YNB supplemented with 2% dextrose. The main large culture of the experimental strains was then partitioned into separate containers and subjected to experimental conditions where indicated. At 10 min before the indicated time point, a 50-ml volume of culture was taken then centrifuged and the resulting supernatant was removed from the yeast pellet. The pellet was then suspended in 5 ml YNB-2% dextrose supplemented for 10 min with either 150 μg/ml puromycin (catalog no. P8833; Sigma), hydrogen peroxide, 100 μg/ml cycloheximide, or a combination thereof as indicated in the figure or figure legend. A brief puromycin exposure time was used to limit the detrimental effects of aberrant protein buildup that occurs due to the early termination of nascent polypeptide chains. After 10 min of puromycin incorporation, lysate was acquired using the same method as described above.

### Flow cytometry-Live/Dead staining.

Flow cytometry data were acquired using a BD LSRFortessa Cell Analyzer. Yeast were grown to exponential phase in minimal medium supplemented with 2% dextrose. Cultures were then treated as outlined in Fig. 6D. After the 2-h incubation step, cultures were washed with 1× phosphate-buffered saline (PBS) and suspended in 50 μl 1× PBS. The Live-Dye yeast stain (catalog no. 31062; Biotium, Fremont, CA) protocol was performed as described by the manufacturer, with the dead and thiazole orange stains incubated with cultures at room temperature for 30 min. Yeast were then fixed in a final concentration of 4% formaldehyde overnight at 4°C. Samples were then diluted with a 3-ml volume of 4% formaldehyde-1× PBS solution, which was used for sample input. The fluorescein isothiocyanate (FITC) channel was used to detect thiazole orange, while the Texas Red channel was used to detect dead yeast (42).

### Quantification and statistical analysis.

Statistical analyses were performed using GraphPad Prism (version 6.05) software. Statistical analyses for stability data were performed by determining the least-squares fit of one-phase exponential decay nonlinear regression with GraphPad Prism software. Significance between curves was detected by a sum-of-squares F test, with a *P* value of <0.05 determining that the data fall on separate regression lines and therefore exhibit different rates of decay. Statistical analysis to compare mRNA abundances between the wild type and the *gcn2*Δ mutant was performed using the Student *t* test.

## References

[1] Rajasingham R, Smith RM, Park BJ, Jarvis JN, Govender NP, Chiller TM, Denning DW, Loyse A, Boulware DR 2017 Global burden of disease of HIV-associated cryptococcal meningitis: an updated analysis. Lancet Infect Dis 17:873–881. doi:10.1016/S1473-3099(17)30243-8. 28483415 PMC5818156

[2] Classen A, Lloberas J, Celada A 2009 Macrophage activation: classical versus alternative. Methods Mol Biol 531:29–43. doi:10.1007/978-1-59745-396-7_3. 19347309

[3] Leopold Wager CM, Hole CR, Wozniak KL, Olszewski MA, Wormley FL Jr 2014 STAT1 signaling is essential for protection against *Cryptococcus neoformans* infection in mice. J Immunol 193:4060–4071. doi:10.4049/jimmunol.1400318. 25200956 PMC4185263

[4] Voelz K, Lammas DA, May RC 2009 Cytokine signaling regulates the outcome of intracellular macrophage parasitism by *Cryptococcus neoformans*. Infect Immun 77:3450–3457. doi:10.1128/IAI.00297-09. 19487474 PMC2715691

[5] Morano KA, Grant CM, Moye-Rowley WS 2012 The response to heat shock and oxidative stress in *Saccharomyces cerevisiae*. Genetics 190:1157–1195. doi:10.1534/genetics.111.128033. 22209905 PMC3316637

[6] Upadhya R, Campbell LT, Donlin MJ, Aurora R, Lodge JK 2013 Global transcriptome profile of *Cryptococcus neoformans* during exposure to hydrogen peroxide induced oxidative stress. PLoS One 8:e55110. doi:10.1371/journal.pone.0055110. 23383070 PMC3557267

[7] Shenton D, Smirnova JB, Selley JN, Carroll K, Hubbard SJ, Pavitt GD, Ashe MP, Grant CM 2006 Global translational responses to oxidative stress impact upon multiple levels of protein synthesis. J Biol Chem 281:29011–29021. doi:10.1074/jbc.M601545200. 16849329

[8] Mascarenhas C, Edwards-Ingram LC, Zeef L, Shenton D, Ashe MP, Grant CM 2008 Gcn4 is required for the response to peroxide stress in the yeast *Saccharomyces cerevisiae*. Mol Biol Cell 19:2995–3007. doi:10.1091/mbc.e07-11-1173. 18417611 PMC2441660

[9] Muhlrad D, Parker R 1992 Mutations affecting stability and deadenylation of the yeast *MFA2* transcript. Genes Dev 6:2100–2111. doi:10.1101/gad.6.11.2100. 1427074

[10] Decker CJ, Parker R 1993 A turnover pathway for both stable and unstable mRNAs in yeast: evidence for a requirement for deadenylation. Genes Dev 7:1632–1643. doi:10.1101/gad.7.8.1632. 8393418

[11] van Dijk E, Cougot N, Meyer S, Babajko S, Wahle E, Séraphin B 2002 Human Dcp2: a catalytically active mRNA decapping enzyme located in specific cytoplasmic structures. EMBO J 21:6915–6924. doi:10.1093/emboj/cdf678. 12486012 PMC139098

[12] Hsu CL, Stevens A 1993 Yeast cells lacking 5′→3′ exoribonuclease 1 contain mRNA species that are poly(A) deficient and partially lack the 5′ cap structure. Mol Cell Biol 13:4826–4835. doi:10.1128/mcb.13.8.4826. 8336719 PMC360109

[13] Chan LY, Mugler CF, Heinrich S, Vallotton P, Weis K 2018 Non-invasive measurement of mRNA decay reveals translation initiation as the major determinant of mRNA stability. Elife 7:32536. doi:10.7554/eLife.32536. PMC6152797 30192227

[14] Grant CM 2011 Regulation of translation by hydrogen peroxide. Antioxid Redox Signal 15:191–203. doi:10.1089/ars.2010.3699. 21126188

[15] Schmidt EK, Clavarino G, Ceppi M, Pierre P 2009 SUnSET, a nonradioactive method to monitor protein synthesis. Nat Methods 6:275–277. doi:10.1038/nmeth.1314. 19305406

[16] Unbehaun A, Borukhov SI, Hellen CU, Pestova TV 2004 Release of initiation factors from 48S complexes during ribosomal subunit joining and the link between establishment of codon-anticodon base-pairing and hydrolysis of eIF2-bound GTP. Genes Dev 18:3078–3093. doi:10.1101/gad.1255704. 15601822 PMC535918

[17] Lu PD, Harding HP, Ron D 2004 Translation reinitiation at alternative open reading frames regulates gene expression in an integrated stress response. J Cell Biol 167:27–33. doi:10.1083/jcb.200408003. 15479734 PMC2172506

[18] Baird TD, Wek RC 2012 Eukaryotic initiation factor 2 phosphorylation and translational control in metabolism. Adv Nutr 3:307–321. doi:10.3945/an.112.002113. 22585904 PMC3649462

[19] Wu CC, Zinshteyn B, Wehner KA, Green R 2019 High-resolution ribosome profiling defines discrete ribosome elongation states and translational regulation during cellular stress. Mol Cell 73:959–970.e5. doi:10.1016/j.molcel.2018.12.009. 30686592 PMC6411040

[20] Matsuo R, Kubota H, Obata T, Kito K, Ota K, Kitazono T, Ibayashi S, Sasaki T, Iida M, Ito T 2005 The yeast eIF4E-associated protein Eap1p attenuates GCN4 translation upon TOR-inactivation. FEBS Lett 579:2433–2438. doi:10.1016/j.febslet.2005.03.043. 15848184

[21] Hanson G, Coller J 2018 Codon optimality, bias and usage in translation and mRNA decay. Nat Rev Mol Cell Biol 19:20–30. doi:10.1038/nrm.2017.91. 29018283 PMC6594389

[22] Vattem KM, Wek RC 2004 Reinitiation involving upstream ORFs regulates ATF4 mRNA translation in mammalian cells. Proc Natl Acad Sci U S A 101:11269–11274. doi:10.1073/pnas.0400541101. 15277680 PMC509193

[23] Thompson SR, Gulyas KD, Sarnow P 2001 Internal initiation in Saccharomyces cerevisiae mediated by an initiator tRNA/eIF2-independent internal ribosome entry site element. Proc Natl Acad Sci U S A 98:12972–12977. doi:10.1073/pnas.241286698. 11687653 PMC60809

[24] Neafsey DE, Galagan JE 2007 Dual modes of natural selection on upstream open reading frames. Mol Biol Evol 24:1744–1751. doi:10.1093/molbev/msm093. 17494029

[25] Janbon G, Ormerod KL, Paulet D, Byrnes EJ, Yadav V, Chatterjee G, Mullapudi N, Hon C-C, Billmyre RB, Brunel F, Bahn Y-S, Chen W, Chen Y, Chow EWL, Coppée J-Y, Floyd-Averette A, Gaillardin C, Gerik KJ, Goldberg J, Gonzalez-Hilarion S, Gujja S, Hamlin JL, Hsueh Y-P, Ianiri G, Jones S, Kodira CD, Kozubowski L, Lam W, Marra M, Mesner LD, Mieczkowski PA, Moyrand F, Nielsen K, Proux C, Rossignol T, Schein JE, Sun S, Wollschlaeger C, Wood IA, Zeng Q, Neuvéglise C, Newlon CS, Perfect JR, Lodge JK, Idnurm A, Stajich JE, Kronstad JW, Sanyal K, Heitman J, Fraser JA, Cuomo CA, Dietrich FS 2014 Analysis of the genome and transcriptome of *Cryptococcus neoformans var. grubii* reveals complex RNA expression and microevolution leading to virulence attenuation. PLoS Genet 10:e1004261. doi:10.1371/journal.pgen.1004261. 24743168 PMC3990503

[26] Tomalin LE, Day AM, Underwood ZE, Smith GR, Pezze PD, Rallis C, Patel W, Dickinson BC, Bahler J, Brewer TF, Chang CJL, Shanley DP, Veal EA 2016 Increasing extracellular H_2_O_2_ produces a bi-phasic response in intracellular H_2_O_2_, with peroxiredoxin hyperoxidation only triggered once the cellular H_2_O_2_-buffering capacity is overwhelmed. Free Radic Biol Med 95:333–348. doi:10.1016/j.freeradbiomed.2016.02.035. 26944189 PMC4891068

[27] Missall TA, Lodge JK 2005 Thioredoxin reductase is essential for viability in the fungal pathogen *Cryptococcus neoformans*. Eukaryot Cell 4:487–489. doi:10.1128/EC.4.2.487-489.2005. 15701811 PMC549343

[28] Picazo C, Matallana E, Aranda A 2018 Yeast thioredoxin reductase Trr1p controls TORC1-regulated processes. Sci Rep 8:16500. doi:10.1038/s41598-018-34908-4. 30405153 PMC6220292

[29] Missall TA, Lodge JK 2005 Function of the thioredoxin proteins in *Cryptococcus neoformans* during stress or virulence and regulation by putative transcriptional modulators. Mol Microbiol 57:847–858. doi:10.1111/j.1365-2958.2005.04735.x. 16045626

[30] Fomenko DE, Koc A, Agisheva N, Jacobsen M, Kaya A, Malinouski M, Rutherford JC, Siu KL, Jin DY, Winge DR, Gladyshev VN 2011 Thiol peroxidases mediate specific genome-wide regulation of gene expression in response to hydrogen peroxide. Proc Natl Acad Sci U S A 108:2729–2734. doi:10.1073/pnas.1010721108. 21282621 PMC3041109

[31] Ogusucu R, Rettori D, Munhoz DC, Netto LES, Augusto O 2007 Reactions of yeast thioredoxin peroxidases I and II with hydrogen peroxide and peroxynitrite: rate constants by competitive kinetics. Free Radic Biol Med 42:326–334. doi:10.1016/j.freeradbiomed.2006.10.042. 17210445

[32] Banerjee D, Bloom AL, Panepinto JC 2016 Opposing PKA and Hog1 signals control the post-transcriptional response to glucose availability in *Cryptococcus neoformans*. Mol Microbiol 102:306–320. doi:10.1111/mmi.13461. 27387858 PMC5055444

[33] Leipheimer J, Bloom ALM, Baumstark T, Panepinto JC 2018 CNBP homologues Gis2 and Znf9 interact with a putative G-quadruplex-forming 3′ untranslated region, altering polysome association and stress tolerance in *Cryptococcus neoformans*. mSphere 3:e00201-18. doi:10.1128/mSphere.00201-18. 30089646 PMC6083090

[34] Bloom AL, Solomons JT, Havel VE, Panepinto JC 2013 Uncoupling of mRNA synthesis and degradation impairs adaptation to host temperature in *Cryptococcus neoformans*. Mol Microbiol 89:65–83. doi:10.1111/mmi.12258. 23659661 PMC4103160

[35] Wallace E, Maufrais C, Sales-Lee J, Tuck L, de Oliveira L, Feuerbach F, Moyrand F, Natarajan P, Madhani HD, Janbon G 2019 Start codon context controls translation initiation in the fungal kingdom. bioRxiv doi:10.1101/654046. PMC7049704 32020195

[36] Weids AJ, Ibstedt S, Tamas MJ, Grant CM 2016 Distinct stress conditions result in aggregation of proteins with similar properties. Sci Rep 6:24554. doi:10.1038/srep24554. 27086931 PMC4834537

[37] Jamar NH, Kritsiligkou P, Grant CM 2018 Loss of mRNA surveillance pathways results in widespread protein aggregation. Sci Rep 8:3894. doi:10.1038/s41598-018-22183-2. 29497115 PMC5832753

[38] Thommen M, Holtkamp W, Rodnina MV 2017 Co-translational protein folding: progress and methods. Curr Opin Struct Biol 42:83–89. doi:10.1016/j.sbi.2016.11.020. 27940242

[39] Panepinto J, Liu LD, Ramos J, Zhu XD, Valyi-Nagy T, Eksi S, Fu JM, Jaffe HA, Wickes B, Williamson PR 2005 The DEAD-box RNA helicase Vad1 regulates multiple virulence-associated genes in *Cryptococcus neoformans*. J Clin Invest 115:632–641. doi:10.1172/JCI23048. 15765146 PMC1051994

[40] Havel VE, Wool NK, Ayad D, Downey KM, Wilson CF, Larsen P, Djordjevic JT, Panepinto JC 2011 Ccr4 promotes resolution of the endoplasmic reticulum stress response during host temperature adaptation in *Cryptococcus neoformans*. Eukaryot Cell 10:895–901. doi:10.1128/EC.00006-11. 21602483 PMC3147416

[41] Kaur JN, Panepinto JC 2016 Morphotype-specific effector functions of *Cryptococcus neoformans PUM1*. Sci Rep 6:23638. doi:10.1038/srep23638. 27008977 PMC4806291

[42] Zhu HP, Clark SM, Benson SC, Rye HS, Glazer AN, Mathies RA 1994 High-sensitivity capillary electrophoresis of double-stranded DNA fragments using monomeric and dimeric fluorescent intercalating dyes. Anal Chem 66:1941–1948. doi:10.1021/ac00085a004. 8067520

