# Supplementary figures and images for: Translational Regulation Promotes Oxidative Stress Resistance in the Human Fungal Pathogen Cryptococcus neoformans
Source: mBio. 2019 Nov 12;10(6):e02143-19. doi: 10.1128/mBio.02143-19 (PMC6851278; doi:10.1128/mBio.02143-19)

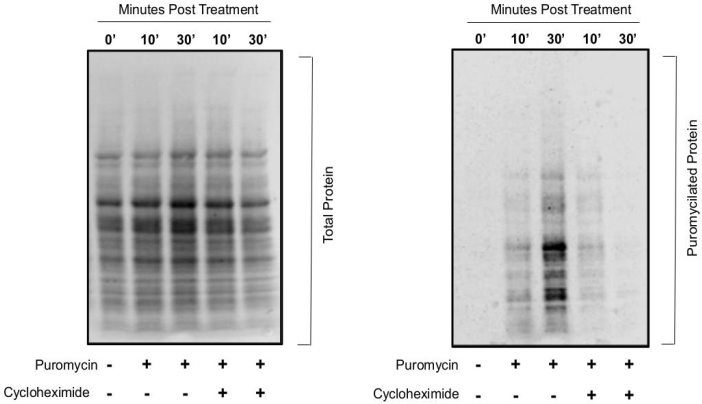

Supplement: FIG S1 [file mBio.02143-19-sf001.tif]

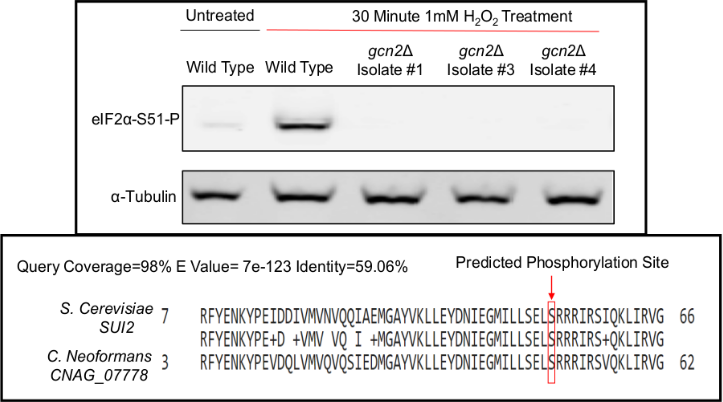

Supplement: FIG S2 [file mBio.02143-19-sf002.tif]

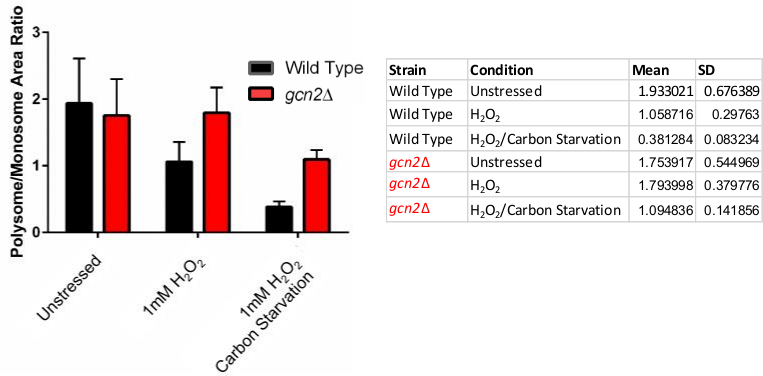

Supplement: FIG S3 [file mBio.02143-19-sf003.tif]

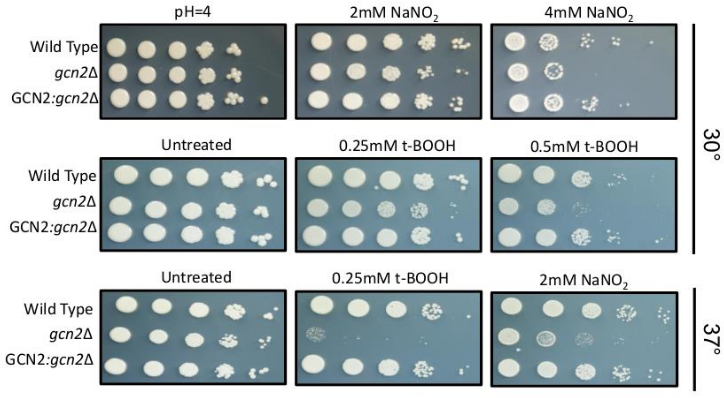

Supplement: FIG S4 [file mBio.02143-19-sf004.tif]

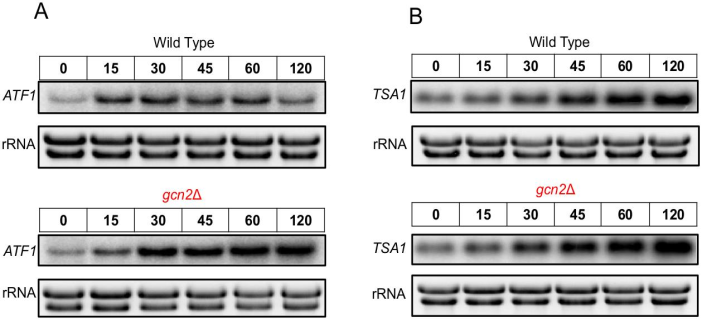

Supplement: FIG S5 [file mBio.02143-19-sf005.tif]

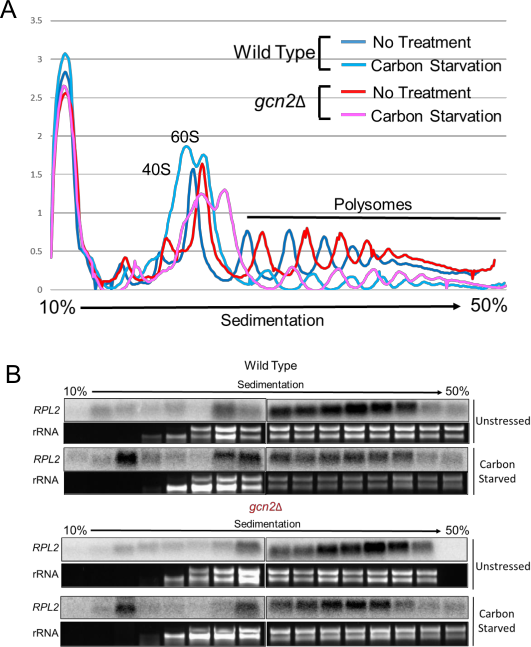

Supplement: FIG S6 [file mBio.02143-19-sf006.tif]

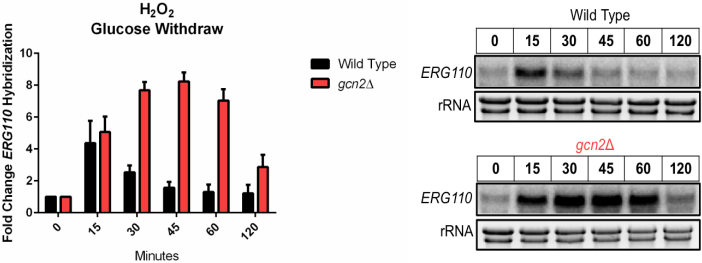

Supplement: FIG S7 [file mBio.02143-19-sf007.tif]
