# Supplementary material for: Translational Regulation Promotes Oxidative Stress Resistance in the Human Fungal Pathogen Cryptococcus neoformans
Source: mBio. 2019 Nov 12;10(6):e02143-19. doi: 10.1128/mBio.02143-19 (PMC6851278; doi:10.1128/mBio.02143-19)
Supplement: TABLE S1 [file mBio.02143-19-st001.pdf]

Oligonucleotide Name	Oligonucleotide Sequence
F-GCN2upKO-Spe1	taataaactagtAGCATTGGATACTACCGAGG
R-GCN2upKO-BamH1	taataaggatccGTCTTTGGTGAGTCTCCG
F-GCN2downKO-Sac1	taataagagctcGCTCATGATCTTTGTACC
R-GCN2downKO-Spe1	taataaactagtCGACCTCTTGACCACTCTC
F-GCN2-Apa1	taataagggcccACTCATCAAAGCAGGTCCG
R-GCN2-Apa1	taataagggcccTATGTTCACCATCGCCCT
RPL2-Northern-fwd	TATCTTCAAGTCCCACACCCACC
RPL2-Northern-rev	CAACATCCCATCCCCACTCC
ERG110-Northern-fwd	CCTTTCCACCGTTTACCTG
ERG110-Northern-rev	CTATCTCAGTTGCTCGCTTG
TRR1-Northern-fwd	CGAGAACTTCCCTGGTTTC
TRR1-Northern-Rev	GTATCCATCGCTGTCAAGC
ATF1-Northern-fwd	CATAACCATCTGCCCAACTTTG
ATF1-Northern-rev	TTCCCGCTTTCCTTGCTCTC
TSA1-Northern-fwd	AGGTCATCTGTGTCTCCACC
TSA1-Northern-rev	CAACATTGCGTTCGCCTATG
GCN2-Northern-fwd	TGTCGGACTCAATGACTGAAC
GCN2-Northern-rev	CCTGTTGGAAGAGAGAAGACTG

Table S1 Oligonucleotide Sequences. Capital letters indicate portion of the primer that hybridizes to the genome. Restriction Sites are underlined
